# The Integral Role of Cardiac Imaging in the Electrophysiology Field

**DOI:** 10.19102/icrm.2025.16111

**Published:** 2025-11-15

**Authors:** Sunu Budhi Raharjo, Andi Muhammad Zharfan

**Affiliations:** 1 Division of Arrhythmia, Department of Cardiology and Vascular Medicine, Faculty of Medicine, Universitas Indonesia/National Cardiovascular Center Harapan Kita, Jakarta, Indonesia; 2 Master of Public Health, Monash University Indonesia, South Tangerang, Indonesia

**Keywords:** Atrial fibrillation, cardiac electrophysiology, electrophysiological procedure, imaging, ventricular arrhythmia

## Abstract

Cardiac imaging is crucial in the electrophysiology field, not only as a diagnostic tool but also to expand and guide interventional cardiac electrophysiology procedures. With this consideration in mind, this article aims to review the critical role of cardiac imaging in stratifying patients’ risk profiles and assisting the electrophysiological procedure in various scenarios. This article also highlights the future direction within the cardiac electrophysiology field with digital twins, which incorporate cardiac imaging and clinical data to build physiologically accurate cardiac replicas to assist electrophysiological procedural planning.

## Introduction

Cardiac electrophysiology is a fast-growing field that involves identifying and treating arrhythmic disorders in both invasive and non-invasive manners. Cardiac imaging has an essential role in the electrophysiology field due to the realization that defining cardiac anatomy and its function is crucial for the successful diagnosis and subsequent management of arrhythmia.^1^ Therefore, this article aims to review the role of cardiac imaging based on current evidence in three common scenarios within the cardiac electrophysiology field: non-invasive assessment, assisting invasive electrophysiology procedures, and guiding cardiac device implantation.

## Non-invasive assessment of cardiac electrophysiology

Non-invasive assessment refers to imaging techniques used to obtain information related to cardiac structure and function through relatively safe methods as opposed to invasive techniques that carry significant peri- and post-procedural risks.^2^ This assessment is critical in the cardiac electrophysiology field, as the detailed information provided is sufficient to detect abnormalities within the myocardial structure.^1,2^ This section will discuss the importance of non-invasive assessment in diagnosing and managing arrhythmogenic cardiomyopathy (ACM), which is one of the life-threatening cardiac diseases.

### The role of cardiac imaging in diagnosing arrhythmogenic cardiomyopathy

ACM is an inherent cardiomyopathy characterized by the infiltration of fibrofatty tissue within the myocardium, which is significantly associated with sustained monomorphic ventricular tachycardia (VT) and, subsequently, sudden cardiac death (SCD).^3^ Electrocardiography (ECG) is commonly used as a first-line modality to identify patients with ACM based on the presence of T-wave inversion or epsilon wave in the precordial leads.^3^ However, these observations can also be attributed to physiological adaptation, particularly among young athletes.^4^ Thus, adjunctive tools beyond ECG are useful to better discriminate patients with ACM.

**Figure 1:**
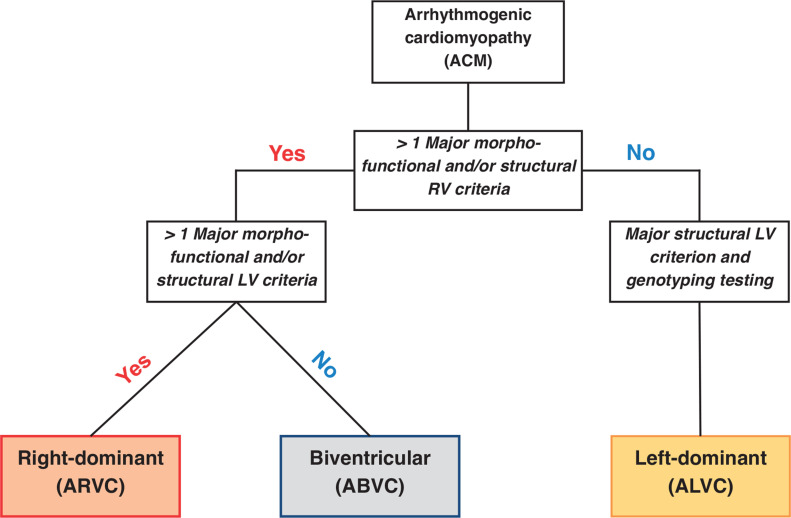
Diagnostic flowchart of arrhythmogenic cardiomyopathy. Adapted from Corrado et al.^3^ *Abbreviations:* ABVC, arrhythmogenic biventricular cardiomyopathy; ALVC, arrhythmogenic left ventricular cardiomyopathy; ARVC, arrhythmogenic right ventricular cardiomyopathy; LV, left ventricular; RV, right ventricular.

The diagnosis of ACM is based on a multiparametric approach that is grouped into six categories: morpho-functional abnormalities, structural myocardial changes, ECG alterations (repolarization and depolarization), ventricular arrhythmias (VAs), and genetic factors.^3^ Each category is further classified into major and minor criteria based on their specificity in distinguishing ACM from other diseases.^5^ A definitive diagnosis of ACM is established by the presence of two major criteria, one major and two minor criteria, or four minor criteria from different categories.^5^ The diagnosis of ACM requires at least one criterion (either major or minor) from category I (ie, morpho-functional abnormalities) or category II (ie, structural abnormalities) to be fulfilled, which can only be assessed using cardiac imaging modalities **(Figure 1)**.^5^

Echocardiography is considered the first-line modality for conducting the initial evaluation of ACM due to its wide availability and accessibility. However, owing to its limited capacity to visualize the complex geometry of the atrial and ventricular chambers, advanced cardiac imaging techniques (eg, cardiac magnetic resonance [CMR], cardiac computed tomography angiography [CTA]) have emerged as promising alternatives for detecting morpho-functional abnormalities associated with ACM.^3^ Furthermore, CMR plays a key role in identifying the fibrofatty tissue replacement of the myocardium, which is the hallmark lesion of ACM. Therefore, advanced cardiac imaging, including CMR and CTA, is crucial for identifying major criteria for ACM.^5^

Despite the availability of cardiac imaging technologies, the diagnostic aspect of ACM still poses a significant challenge. The difficulties are pronounced when assessing arrhythmogenic left ventricular (LV) cardiomyopathy, which exhibits a similar clinical presentation (the so-called “hot phase”) and image findings (eg, inferolateral mid-epicardial late-gadolinium enhancement [LGE] involvement) to myocarditis.^6^ Therefore, in addition to the clinical findings and cardiac imaging, genetic testing is still required to confirm the diagnosis of ACM.

### Cardiac imaging and the management of arrhythmogenic cardiomyopathy

The management of ACM focuses on early intervention to prevent premature death or SCD through insertion of an implantable cardioverter-defibrillator (ICD).^7^ However, the decision for ICD implantation should weigh the potential risks, such as inappropriate shock and lead malfunction, which commonly occur among young populations and athletes.^8^ Therefore, stratifying the risk–benefit ratio of ICD is an integral part of managing ACM patients. Cardiac imaging, especially CMR, has been considered a valuable tool for stratifying the patient’s risk of SCD by evaluating the degree of myocardial (ie, right ventricular and LV) dysfunction and myocardial scar, which is positively associated with the risk of VA and SCD.^9^ Furthermore, considering the progressive nature of ACM, cardiac imaging should be re-evaluated annually to dynamically track the risk of VA and SCD.

## Assisting invasive electrophysiology procedures

The cardiac electrophysiology field relies heavily on cardiac imaging to plan the appropriate strategies and stratify the patients who will benefit from the invasive electrophysiological procedure. Cardiac imaging plays a critical role in assisting catheter ablation for atrial fibrillation (AF) and VA, which will be explained in the following section.

### Catheter ablation for atrial fibrillation

AF is the most common form of arrhythmia, with a prevalence of approximately 60 million cases, which is expected to increase in the upcoming years with the growth of aging populations.^10^ The presence of AF is also associated with adverse events, including stroke, myocardial infarction, heart failure (HF), and death.^10^ Catheter ablation is established as a therapy option that has been recommended to reduce AF burden and its progression, especially for patients with drug-resistant AF.^11^ The ablative procedure can be delivered through various methods, such as radiofrequency catheter ablation, cryoablation, and pulsed-field ablation.^11^ Although their numbers are low, the ablative procedure is associated with various adverse outcomes, such as pulmonary vein (PV) stenosis, ischemic stroke, phrenic nerve palsy, and atrioesophageal fistula.^12^ Moreover, nearly half of the patients who undergo AF ablation will develop recurrent AF in the future.^13^ Therefore, careful patient selection and comprehensive procedural planning are essential for achieving favorable outcomes of AF ablation. Performing cardiac imaging is pivotal for identifying atrial abnormalities associated with poor outcomes of AF ablation, which is crucial to optimize patient selection. The following sections will further explore the role of imaging modalities that are commonly used to evaluate patients who plan to undergo AF ablation procedures, including echocardiography, CTA, and CMR.

### The role of echocardiography in atrial fibrillation ablation

Echocardiography is an imaging modality that is used to evaluate the structural abnormalities of the heart, stratify the risk profile during the peri-procedural phase, and assist AF ablation. There are three types of echocardiography that are widely used for examining patients with cardiac disease, which are transthoracic echocardiography (TTE), transesophageal echocardiography (TEE), and intracardiac echocardiography (ICE).

TTE has been recommended as the primary imaging modality to assess patients with AF due to its availability and accessibility.^11^ TTE is commonly used for the early detection of structural and functional abnormalities, such as atrial enlargement and LV ejection fraction (LVEF), to determine whether additional imaging is needed for better accuracy.^11^ However, the use of TTE during procedural planning is restricted due to limited tissue characterization and a narrow field of view.^14^ Thus, another modality is required to better visualize the anatomical structure of the left atrium.

**Figure 2:**
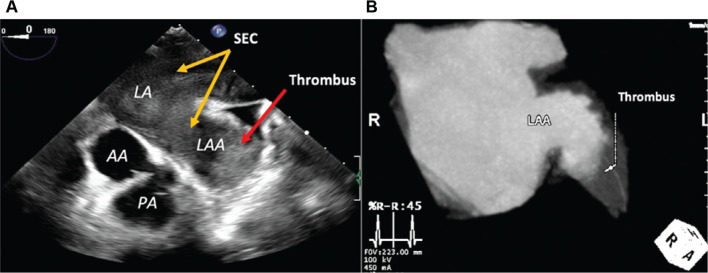
Differences in thrombus detection by transesophageal echocardiography **(A)** and cardiac computed tomography angiography **(B)**. Transesophageal echocardiography directly shows the presence of thrombus and spontaneous echo contrast in the left atrial appendage and left atrium, respectively **(A)**, whereas cardiac computed tomography angiography shows contrast filling defect (thrombus) **(B)**. *Abbreviations:* AA, aorta ascendens; LA, left atrium; LAA, left atrial appendage; PA, pulmonary artery; SEC, spontaneous echo contrast.

**Figure 3:**
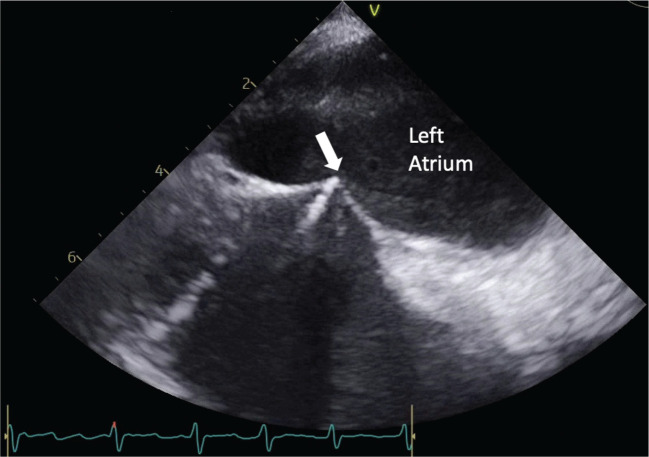
Two-dimensional transesophageal echocardiography guiding the transseptal puncture system toward the central region of the atrial septum. The white arrow indicates the tenting position of the transseptal puncture.

TEE is a semi-invasive imaging modality that is capable of visualizing the cardiac structures and functions that are poorly visible on TTE. In the context of AF ablation, TEE is used for detecting atrial thrombus and assisting the transseptal puncture (TSP) procedure. TEE is considered the gold standard for detecting thrombus and spontaneous echo contrast, which is a precursor of thrombus within the atrial structure **(Figure 2A)**.^11^ Identifying a thrombus is crucial, as the thrombus can dislodge and cause ischemic stroke following AF ablation. A study by Tanaka et al. demonstrated that the intracardiac thrombi detected by TEE increased the risk of AF-associated stroke events by three-fold.^15^ Therefore, TEE is an important imaging modality for detecting the presence of thrombus, especially for patients who have a high risk of stroke, such as those with advanced age, persistent AF (PAF), or an underlying structural heart disease (SHD).^16^ TEE can also be used to improve the safety of the TSP procedure **(Figure 3)**. In brief, the TSP procedure is done to provide access for the catheter to the left atrium to perform the AF ablative procedure. TSP is commonly performed under fluoroscopy guidance to locate the optimal position to conduct the procedure. Although fluoroscopy-guided TSP has a reasonable safety profile, it still carries a significant risk of life-threatening complications, including pericardial effusion and pericardial tamponade.^17^ Thus, incorporating TEE can improve the accuracy and help to mitigate the aforementioned risk. This concept was verified through a study by Katov et al., which confirmed that incorporating TEE, in addition to fluoroscopy, significantly improved TSP localization to the optimal area and prevented dangerous positioning of the TSP.^17^

ICE can also be used as an additional imaging modality to provide real-time monitoring of the catheter position relative to the PV ostium.^18^ ICE ensures an adequate catheter contact with the tissue during the ablation procedure, which translates to optimization of catheter energy delivery and improved procedural efficacy.^19^ In the context of TSP, the use of ICE significantly reduces the fluoroscopy exposure, as it directly visualizes areas that are not seen by fluoroscopy.^18^ However, the use of ICE is severely limited, especially in low- to middle-income countries, as the use of ICE is associated with higher procedural costs.

### The role of cardiac computed tomography angiography and cardiac magnetic resonance in atrial fibrillation ablation

Advanced imaging modalities such as CTA and CMR have emerged as reliable alternatives to TEE to minimize patient discomfort, as the invasive nature of TEE exposes the patient to procedural risks, including esophageal injury.^20^ According to several studies, CTA and/or CMR have been proven beneficial for visualizing atrial abnormalities that are associated with poor procedural outcomes of AF ablation, such as atrial thrombi, atrial enlargement, atrial fibrosis, and PV structures.

Atrial mechanical dysfunction during AF is associated with the development of atrial thrombi, which often leads to the occurrence of stroke. Although TEE is considered the gold standard in detecting atrial thrombi, a meta-analysis by Yu et al. suggested the comparable sensitivity and specificity of CTA to TEE in ruling out atrial thrombi^21^ **(Figure 2B)**. A study by Kitkungvan et al. revealed the efficacy of CMR in detecting atrial thrombi with high sensitivity and specificity (100% and 99.2%, respectively).^22^ Thus, CTA and/or CMR can be used as an alternative to detect the presence of atrial thrombi.

The self-perpetuating nature of AF is associated with structural remodeling, which is often characterized by the change in atrial size^23^ and atrial wall thickness (AWT).^24^ Atrial enlargement has been known as an indicator for atrial remodeling, with the left atrial volume index (LAVI) constituting a common parameter for quantifying the change in atrial size.^23^ Although the LAVI can be measured by two-dimensional (2D) echocardiography, the dependence of the echocardiography on the quality of acoustic windows limits its ability to accurately assess the atrial structure. A recent study by Alajaji et al. suggested that CTA has better accuracy in measuring LAVI compared to echocardiography.^25^ The association between LAVI and AF recurrence following AF ablation has been demonstrated in a study by Putrinarita et al., which shows that the prognostic value of LAVI, measured by CTA, predicts AF recurrence following catheter ablation.^26^

Atrial fibrosis is another hallmark of atrial remodeling in AF patients. The presence of atrial fibrosis is important, as it is capable of altering the myocardial voltage and effective refractory period, leading to an increase in susceptibility to perpetuate AF.^27^ This theory is supported by Marrouche et al., who reported that the atrial fibrosis degree is associated with the AF recurrence rate post-ablation.^28^ Echocardiography offers a limited image resolution, leading to a less comprehensive assessment of substrate characteristics. LGE allows precise characterization of atrial fibrosis through CMR, as the kinetics of gadolinium contrast washout in myocardial fibrosis are much slower than in normal tissue.^29^ The precise characterization of atrial fibrosis using LGE-CMR can help the electrophysiologist determine the appropriate management for AF patients.^29^

AF can also influence the thickening of the atrial wall, which potentially hinders the ability of catheter ablation to produce a complete transmural lesion and enhances the risk of AF recurrence.^30^ These findings are important, as the study by Ratnasari et al. suggested that AWT is strongly associated with the risk of AF recurrence after catheter ablation.^31^ Therefore, incorporating the AWT parameter during procedural planning, which can be measured using CTA, can help the electrophysiologist adjust their ablation strategies to improve outcomes.^24^

PV structural variations are another essential aspect that needs to be recognized during procedural planning, as PV acts as the basis of the ablation procedure. Larsen et al. reported that PV variations, such as a left common pulmonary vein, are linked to a higher rate of AF recurrence post-ablation.^32^ These conditions are presumed to be caused by inadequate contact between the myocardium and the ablation catheter.^33^ In addition to the PV variations, it is crucial to recognize the adjacent PV structure (eg, phrenic nerve and esophagus), as it is prone to injury during the ablative procedure.^12^ The superior image resolution of CTA and/or CMR can help identify both a PV structural variation and its adjacent structure and optimize the procedural planning to reduce the risk of adverse events following catheter ablation.

To sum it up, the ability of advanced imaging modalities such as CTA and CMR is not limited to assisting AF ablation procedures but also selecting appropriate patients. In addition, these modalities have also been shown to individualize ablation strategies to further improve the AF ablation outcomes.

### Image integration in atrial fibrillation ablation

**Figure 4:**
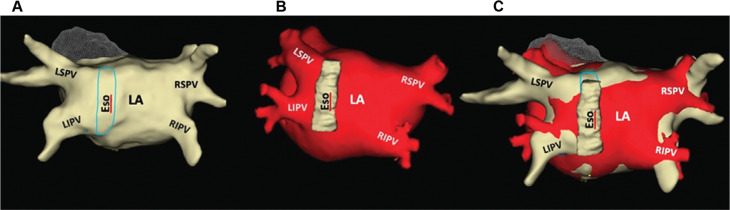
Three-dimensional reconstruction of the left atrium and pulmonary veins created by **(A)** cardiac computed tomography angiography, **(B)** three-dimensional electroanatomic mapping based on the cardiac computed tomography angiography model, and **(C)** merged three-dimensional model. Note that the merged model provides a more realistic anatomy compared with previous models. *Abbreviations:* Eso, esophagus; LA, left atrium; LIPV, left inferior pulmonary vein; LSPV, left superior pulmonary vein; PV, pulmonary vein; RIPV, right inferior pulmonary vein; RSPV, right superior pulmonary vein.

Recent technological advancements enable the integration of preprocedural CTA/CMR data into a three-dimensional (3D) electroanatomic mapping (EAM) system to accurately navigate the catheter during AF ablation procedures **(Figure 4)**. This approach is particularly relevant in the setting of PAF, where the arrhythmogenic substrate of AF frequently emerges from structures beyond PVs, including the left atrial posterior wall, left atrial appendage (LAA), ligament of Marshall, coronary sinus (CS), superior vena cava, and crista terminalis.^34^ Several studies have reported the benefit of integrating various imaging modalities with the EAM system to guide and potentially improve post-procedural outcomes.^35^

Aligning the 3D model into the EAM system remains challenging due to physiological differences between each modality. As a result, the integration of CTA/CMR with EAM can give a misleading impression of the real anatomy during the procedure. This limitation is further demonstrated in a meta-analysis by Mamadli et al., which showed that image integration did not significantly improve AF outcomes.^36^ Despite the conflicting results, the study demonstrated that integrating imaging modalities can improve the safety of ablation procedures by reducing the radiation exposure.

### Catheter ablation of ventricular arrhythmias

VAs are some of the most frequently encountered arrhythmias in clinical settings, characterized by a wide QRS complex tachyarrhythmia and various clinical manifestations from palpitation to SCD.^37^ VAs entail a wide spectrum of rhythm abnormalities, consisting of premature ventricular contractions (PVCs), VT, and ventricular fibrillation, and are commonly categorized based on the presence or absence of SHD. One of the therapeutic options for VAs is catheter ablation, particularly in cases where medical therapy is ineffective.^37^ Cardiac imaging has the potential to aid in the localization and characterization of the arrhythmogenic substrate. Therefore, incorporating cardiac imaging into procedural planning is deemed to be beneficial for selecting patients who will likely benefit from ablative procedures. This section will further explain the role of cardiac imaging in assisting the catheter ablation procedure of VAs in the absence or presence of SHD.

### Cardiac imaging in the absence of structural heart disease

Idiopathic VAs are defined as VAs without any detectable SHD^37^ associated with adverse outcomes,^38^ including ventricular function impairment, HF, and mortality. Idiopathic VAs mainly originate from either the outflow tract, fascicular tissue, or papillary muscles, which can be identified using a QRS axis–based algorithm.^38^ Targeting ablation to this area is challenging due to the anatomical complexity within the outflow tracts that hinders adequate catheter–tissue contact. Thus, cardiac imaging plays a crucial role in accurately localizing the anatomical substrate of the VA and visualizing catheter contact with the cardiac tissue during ablation.

**Figure 5:**
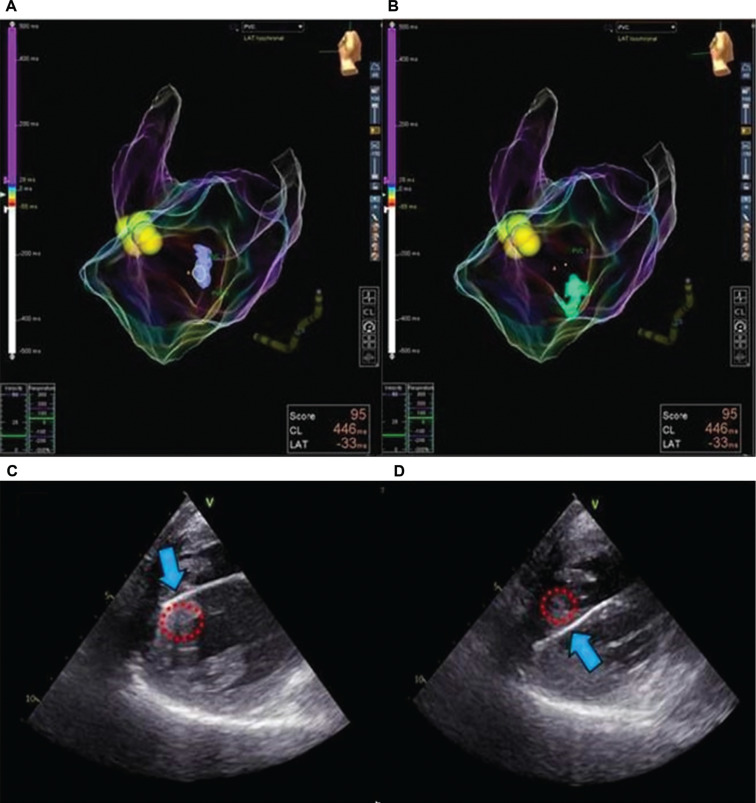
Electroanatomic mapping **(A, C)** and two-dimensional transesophageal echocardiography **(C, D)** guiding catheter ablation of the posterior papillary muscle. Note that transesophageal echocardiography provided additional value to electroanatomic mapping by providing real-time catheter contact with the tip **(C)** and the base **(D)** of the posterior papillary muscle.

CMR is a common imaging modality that is widely used to aid the ablation procedure in idiopathic PVCs, as it has superiority in evaluating scar distribution by using LGE contrast.^37^ Therefore, CMR is considered an ideal primary imaging modality to improve the likelihood of procedural success in the absence of SHD. Additionally, ICE has also emerged as an adjunctive tool for visualizing catheter–tissue contact in real time, especially in papillary muscle arrhythmia.^39^ However, the use of ICE within ablative procedures is associated with higher procedural costs. Aside from those modalities, TEE could also be employed as an alternative for guiding VA ablation procedures based on our experience **(Figure 5)**. However, this finding needs to be validated in a larger cohort.

### Cardiac imaging in the presence of structural heart disease

The scar formation within myocardial tissue is often caused by SHD, either ischemic or non-ischemic cardiomyopathy. The presence of scar tissue is particularly important, as it can induce VAs, particularly VT, through the re-entry mechanism.^37^ Current guidelines suggest the role of catheter ablation as the primary modality to reduce scar-related VT burden by targeting the conduction channel, which can be identified through activation, entrainment, or pace activation mapping.^40^ However, a non-inducibility state or hemodynamic intolerance condition often limits the use of EAM. To overcome this condition, physicians can incorporate data from advanced imaging modalities (eg, CTA and/or CMR) to define the target for catheter ablation. CTA demonstrates superiority over CMR, as it has a higher spatial resolution and provides a more detailed cardiac anatomy (eg, coronary arteries and phrenic nerve), which is useful for reducing the risk of adverse events during an ablative procedure.^41^ However, while CMR has a lower spatial resolution compared to CTA, it offers unique advantages for characterizing scar distribution. The fibrosis substrate in non-ischemic cardiomyopathy is more heterogeneous and complex than that in ischemic cardiomyopathy, as it is often patchy and diffuse, rendering the substrate less pronounced on CTA or EAM.^42^ To overcome this limitation, LGE-CMR can be used to provide a higher image resolution to detect scar distribution within the myocardium. The integration of imaging data from CTA and CMR provides intricate anatomic detail with accurate distribution of the arrhythmogenic substrate within the myocardium and is associated with favorable long-term outcomes, which explains the increasing trend toward their combined use in academic centers.^43^

### Image integration in ventricular arrhythmia ablation

The recent development of scar-segmented software (ie, automatic detection of arrhythmogenic substrate [ADAS] and inHEART) demonstrates its function to delineate potential conduction channels within the ventricular scar, also known as “border zone corridors,” by using CTA or CMR data. The use of ADAS 3D (Adas3D Medical SL, Barcelona, Spain) for procedural guidance of catheter ablation may result in a better VT-free survival rate.^44^ Other software programs (e.g., inHEART; inHEART Medical, Pessac, France) have also emerged, offering promising clinical visualization and analysis of anatomical structures for preprocedural planning and intraprocedural use.

Of note, the main difference between the aforementioned two scar-segmented software programs is the requirement for post-processing.^45^ ADAS 3D requires user input during post-processing, which directly correlates with the user’s expertise. However, ADAS 3D results have a better visualization of the conduction corridors than inHEART results, which makes the former a valuable tool for targeted ablation planning. On the contrary, despite having inferior results relative to ADAS 3D, inHEART offers the advantage of minimal user input during post-processing, which makes this software program more usable by a broader range of clinicians. However, further research is still needed to confirm the efficacy of these two scar-segmented software programs in guiding VA ablation procedures.^44^

## Device implantation

Cardiac device implantation is a common procedure to manage cardiac arrhythmias and arrhythmia adverse events, with over 1 million devices implanted annually worldwide.^46^ Importantly, however, the increasing number of implanted devices corresponds with a rise in both implantation failures and complications. Cardiac imaging has a significant impact on identifying tissue characteristics associated with adverse events and minimizing complication rates. It is also commonly employed in assisting device implantation procedures, such as LAA occlusion (LAAO), cardiac resynchronization therapy (CRT), and left bundle branch area pacing (LBBAP) implantation. Additionally, the imaging process also helps to stratify a patient’s risk profile prior to the device implantation procedure.

### Left atrial appendage occlusion

#### Implantation

One of the major complications of AF is stroke, where AF is associated with a five-fold increased risk of ischemic stroke.^11^ The LAA serves as a major site for thrombus formation in patients with AF due to its complex anatomical properties, predisposing it to blood flow stasis. A thrombus that forms within the LAA structure can dislodge and travel to the brain, causing ischemic stroke. Furthermore, patients who suffer AF-related stroke also experience greater severity and higher mortality rates, contributing to increases in medical and economic burden.^47^ According to the latest European Society of Cardiology and American Heart Association guidelines, LAAO is a promising alternative for patients with AF who have a high risk of bleeding (ie, CHA_2_DS_2_-VASc score ≥ 2 points) and contraindications for long-term oral anticoagulation to prevent thrombus formation.^11^ Cardiac imaging has a pivotal role in ensuring the safety and efficacy of LAAO implantation by visualizing the LAA anatomical structure and evaluating the presence of atrial thrombi.

#### Cardiac imaging in assessing left atrial appendage morphology

LAA is a derivative of the atrial primordium located in the left atrioventricular sulcus and serves as an atrial reservoir in the case of volume overload and neurohormonal modulation. According to a study by Wang et al., CTA or CMR is capable of visualizing the LAA morphology, which further classifies it into four main types: windsock, chicken wing, cactus, and cauliflower.^48^ This visualization is pivotal for assessing the risk of thromboembolic events, as a study by Di Biase et al. demonstrated a link between LAA morphologies and the risk of thromboembolic events.^49^ A meta-analysis by Lupercio et al. also further confirms this observation.^50^

**Figure 6:**
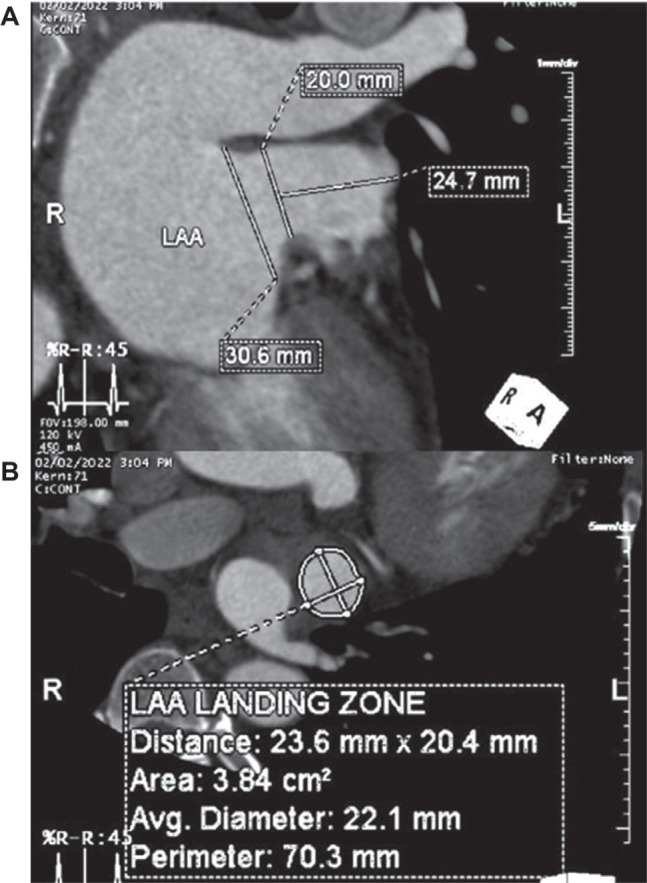
Quantitative measurement of left atrial appendage. Example images showing the view used to measure the ostium **(A)**, landing zone **(A, B)**, and the depth of the left atrial appendage **(A)**.

Beyond LAA morphologies, cardiac imaging can also be employed for measuring LAA orifice and landing zone dimensions to facilitate preprocedural planning of LAAO device implantation. The use of 2D TEE for measuring LAA orifice and landing zone dimensions is common; however, 2D TEE displays limited measurement accuracy due to the complexity of the LAA structure, leading to inappropriate LAAO size and worsening outcomes.^51^ Using advanced cardiac imaging modalities such as CTA^52^ and/or CMR^53,54^ is potentially beneficial, as it provides a more detailed visualization and may accurately measure LAA dimension compared to 2D TEE **(Figure 6)**.

#### Cardiac imaging of a post-procedural left atrial appendage occlusion device implantation

**Figure 7:**
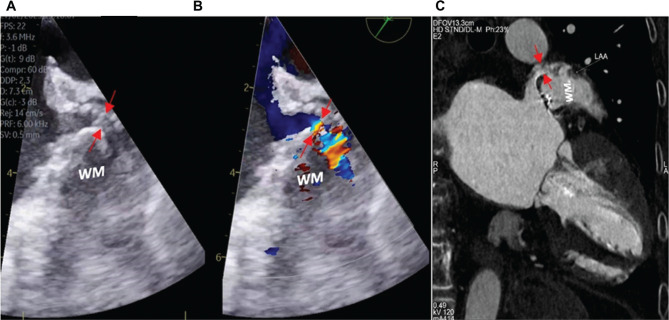
Conventional transesophageal echocardiography **(A)**, color Doppler transesophageal echocardiography **(B)**, and cardiac computed tomography angiography **(C)** depicting a large peri-device leak (red arrows) after Watchman™ device (Boston Scientific, Marlborough, MA, USA) employment.

Conducting a post-procedural evaluation following LAAO implantation is important to detect peri-device leak (PDL), which is defined as a residual communication between the left atrium and the LAA and confers a significant risk of thromboembolic events. This condition is frequently caused by a geometry mismatch between the LAA and the implanted LAAO device. TEE is considered the gold standard for assessing the presence of PDL; however, a recent study reported that CTA presents a comparable sensitivity^55^ in evaluating LAA patency following LAAO implantation **(Figure 7)**.

The formation of the thrombus after LAAO implantation, also known as device-related thrombus (DRT), is a rare incidence associated with adverse clinical outcomes, as it is linked to a four- to five-fold risk of developing a thromboembolic event.^56^ TEE is currently regarded as the gold standard for the diagnosis and evaluation of DRT. Using another imaging modality, such as CTA, is also crucial to evaluate factors associated with DRT formation, such as deep device implantation and LAA orifice width.^56^ Nonetheless, the cumulative evidence shows that the risk of thromboembolic events in patients with DRT is typically based on the composite of patient risk factors (history of stroke, AF type) rather than the presence of DRT itself.

### Cardiac resynchronization therapy

#### Implantation

CRT is a therapeutic modality that received a class I recommendation for symptomatic HF patients with an LVEF of ≤35% and left bundle branch block (LBBB) that causes mechanical dyssynchrony.^57^ Despite its benefits, about one-third to nearly half of CRT recipients remain non-responsive to CRT therapy.^58^ A clinical trial by Singh et al. demonstrated the importance of CRT lead location, which showed that CRT implantation within the apical area of the LV was associated with the risk for HF and death.^59^ Since then, research has been focused on the optimization of CRT lead implantation. Cardiac imaging is crucial to determine the optimal site for CRT lead implantation to increase the responder rate of CRT. This section will explore the role of cardiac imaging modalities and their integration to improve the outcomes of CRT in the general population.

#### Preprocedural imaging of the cardiac resynchronization therapy implantation

Prior to the CRT implantation, cardiac imaging plays a crucial role in determining the optimal CRT lead placement. The specific placement is affected by several factors, which are the CS anatomy, scar tissue, and the mechanical activation of the LV. Understanding coronary venous anatomy is one of the important factors that is paramount in achieving a successful CRT implantation, as the CRT lead will be placed into the CS. Thus, employing cardiac imaging might help to determine the appropriate lead location based on the anatomical variability of the CS and its tributaries.^60^

CS angiography (CSA) is one of the methods that could be used to assess the CS anatomy during preprocedural imaging. However, it resulted in low imaging quality that hindered the full acquisition of anatomical CS data.^61^ Other advanced modalities, such as CTA or CMR, have the ability to better delineate CS anatomy compared to CSA. Specifically, CTA is more commonly used to evaluate CS anatomy, as it has a high level of agreement with CSA and a greater capacity for identifying CS tributaries, which is crucial for deciding on a suitable approach during complex cases.^61^ However, CTA also has a few disadvantages, such as extra cost and radiation exposure, compared to CSA.^62^ Additionally, CMR is also an alternative imaging modality that is capable of delineating coronary venous anatomy with significantly less radiation than CTA. Despite this, CMR has a lower spatial resolution compared to CTA in visualizing the coronary venous tributaries. Therefore, using conventional methods, such as CSA, seems to be reasonable in terms of cost-effectiveness and radiation exposure.^61^

Identification of myocardial fibrosis tissue preceding CRT lead placement is also important, as placing the lead within the scar tissue area might reduce the efficacy of CRT based on the electrophysiological perspective.^60^ This concept is further confirmed in the study by Leyva et al., which showed that lead placement at the area of the scar is associated with a suboptimal response of CRT.^63^ Cardiac imaging, including LGE-CMR, is capable of visualizing myocardial fibrosis, which can improve the outcome of CRT.^61^

Another important factor in the placement of CRT leads is distinguishing the latest mechanically activated LV segment to achieve more synchronized activation of the ventricle to reduce HF burden.^60^ Speckle tracking echocardiography can be used to evaluate the mechanical activation of the LV. The efficacy of this approach was demonstrated in a study by Borgquist et al., which showed that the CRT lead placement based on the last mechanically activated LV segment significantly improves the HF hospitalization rate compared to the standard of care.^64^

#### Multimodal imaging in cardiac resynchronization therapy implantation

The complexities involved in determining optimal CRT lead placement urge the current research to focus on using multimodal imaging approaches for identifying appropriate lead location. However, studies offer conflicting results regarding the benefit of multimodal imaging.^61^ A study by Borgquist et al. reported that integration of echocardiography with other cardiac imaging modalities (CMR and CTA) did not significantly improve the CRT outcomes.^65^ The gap between the theory and real-world application regarding multimodal imaging approaches demonstrated the challenges in determining optimal CRT lead locations, which need to be addressed in future research.

### Left bundle branch area pacing

#### Implantation

LBBAP is a physiological pacing technique that has recently emerged as an alternative to CRT in patients with persistent HF with reduced LVEF and LBBB. It can stimulate myocardial activation beyond the site of block in a physiological manner compared to other physiological pacing strategies (ie, His-bundle pacing [HBP]), resulting in better clinical outcomes.^66,67^ The efficacy of LBBAP has been demonstrated through a study by Chen et al.,^68^ which supported the feasibility of LBBAP as an alternative strategy in CRT non-responder cases. However, myocardial fibrosis poses a significant challenge in screwing the LBBAP lead into the interventricular septum (IVS), as it increases myocardial stiffness.^69^ Therefore, delineating myocardial fibrosis within the IVS is a critical step for selecting suitable patients for the procedure.^70^

As mentioned in the previous section, LGE-CMR can quantify the degree of myocardial fibrosis. The role of LGE-CMR in stratifying the risk of CRT non-response was confirmed in a study by Ali et al., which exhibited that the degree of myocardial fibrosis within the IVS was associated with LBBAP implant failures and LV function impairment in the long term, leading to suboptimal CRT outcomes.^71^ Given this evidence, it can be concluded that cardiac imaging is important during procedural planning to select appropriate patients for LBBAP implantation.

A major challenge in LBBAP placement is achieving adequate lead depth to capture the conduction system without perforating the IVS, as the standard guidance with fluoroscopy and ECG provides no direct visualization of the lead’s position within the myocardial tissue. A recent case series demonstrated the feasibility and utility of using TEE to guide LBBAP implantation, as it provides direct, real-time visualization of the IVS, allowing the operator to guide lead placement and monitor the depth of the LBBAP lead.^72^ ICE also offers similar benefits without the need for general anesthesia, but at a higher cost. Therefore, when managing complex cases, TEE can provide significant value to improve the success rate of LBBAP lead placement.

## Looking forward: digital twin of the heart

The advancement in the software engineering field has enabled the construction of a personalized computational model of the heart, or the so-called “digital twin of the heart,” by integrating various data sources, including imaging findings from CTA or CMR and electrophysiological properties from ECG or EAM.^73^ The digital twin is deemed to be superior compared to conventional models, as it is capable of mimicking the dynamics of cardiac electrophysiological function in a virtual environment. Thus, the development of digital twins within the electrophysiology field is crucial to bring up an opportunity to tailor the treatment based on the patient’s cardiac electrophysiological profile and optimize the outcomes.^73^ The digital twin also holds promising potential to assist electrophysiological procedures such as CRT implantation and VT ablation. A study by Koopsen et al. revealed that the digital twin is capable of predicting the outcomes of CRT based on different pacing modalities (ie, HBP vs. left bundle branch pacing vs. biventricular pacing).^74^ Furthermore, a recent study by Waight et al. demonstrated the efficacy of the digital twin to uncover the VT circuit of the scar-mediated VT, which is critical during the ablation procedure.^75^ However, further research is still required to determine its efficacy in clinical settings.

## Conclusion

The cardiac electrophysiology field heavily relies upon multimodal cardiac imaging to depict accurate cardiac structure and optimize the cardiac electrophysiological procedural strategies. As a non-invasive diagnostic modality, cardiac imaging is important to determine the presence of cardiac abnormalities that were not captured by other modalities, such as scar tissue or functional abnormalities. Furthermore, cardiac imaging is also principal in assisting invasive procedures, such as ablation, by depicting the anatomical structure and stratifying patients’ risk profiles, which is crucial for improving the cost-effectiveness of health care resource utilization. Lastly, in the device implantation procedures, cardiac imaging also plays a significant role in evaluating cardiac structure in detail, which leads to optimal device and patient selection. The advancement of computational modeling to simulate the physiological replica of the patient’s heart unfolds opportunities to unravel the patient’s mechanisms of arrhythmia and individualize procedural strategies using a virtual model, which can significantly improve patient outcomes. Thus, the future of cardiac imaging should focus on the integration of cardiac imaging modalities with artificial intelligence to individualize procedural strategies to increase the effectiveness and efficiency of electrophysiological procedures.
